# Antimicrobial Usage in Animal Production: A Review of the Literature with a Focus on Low- and Middle-Income Countries

**DOI:** 10.3390/antibiotics7030075

**Published:** 2018-08-15

**Authors:** Nguyen V. Cuong, Pawin Padungtod, Guy Thwaites, Juan J. Carrique-Mas

**Affiliations:** 1 Oxford University Clinical Research Unit, 764 Vo Van Kiet, District 5, Ho Chi Minh City, Vietnam; cuongnv@oucru.org (N.V.C.); gthwaites@oucru.org (G.T.); 2 Emergency Center for Transboundary Animal Diseases, Food and Agriculture Organization of the United Nations, Green One UN House Building, 304 Kim Ma, Hanoi, Vietnam; Pawin.Padungtod@fao.org; 3 Centre for Tropical Medicine and Global Health, Nuffield Department of Medicine, Oxford University, Old Road Campus, Headington, Oxford OX3 7BN, UK

**Keywords:** antimicrobial use, livestock, poultry, metrics, pigs, cattle, chickens

## Abstract

Antimicrobial use (AMU) in animal production is a key contributor to antimicrobial resistance (AMR) worldwide. As consumption of animal protein and associated animal production is forecast to increase markedly over coming years in low- and middle-income countries (LMICs), accurate monitoring of AMU has become imperative. We summarized data from 89 scientific studies reporting AMU data in animal production published in English since 1998, identified through the ‘ISI Web of Knowledge’ search engine. The aims were as follows: (a) to describe methodologies and metrics used to quantify AMU; (b) to summarize qualitative (on-farm prevalence of use) and quantitative (amounts of antimicrobial active principle) data, in order to identify food animal species at the highest risk of AMU; and (c) to highlight data gaps from LMICs. Only 17/89 (19.1%) studies were conducted in LMICs. Sixty (67.3%) reported quantitative data use, with ‘daily doses per animal-time’ being the most common metric. AMU was greatest in chickens (138 doses/1000 animal-days [inter quartile range (IQR) 91.1–438.3]), followed by swine (40.2 [IQR 8.5–120.4]), and dairy cattle (10.0 [IQR 5.5–13.6]). However, per kg of meat produced, AMU was highest in swine, followed by chickens and cattle. Our review highlights a large deficit of data from LMICs, and provides a reference for comparison with further surveillance and research initiatives aiming to reduce AMU in animal production globally.

## 1. Introduction

Antimicrobials are used worldwide both in humans and in animals for the prevention and treatment of infectious diseases [1]. In addition, in some countries, antimicrobials are used in animal farming as growth promoters [2]. A correlation between antimicrobial use (AMU) and antimicrobial resistance (AMR) in animal production has been firmly established from observational studies [3,4], country AMU/AMR surveillance data [5,6], and statistical meta-analyses [7]. Increased levels of AMR have a negative impact on livestock production, either by reducing farm productivity, or by higher costs of disease treatment [8]. However, much of the impetus for monitoring AMU/AMR in animal production has stemmed from an emerging scientific consensus supporting the contribution of AMU/AMR in animal production on the overall burden of AMR in humans [9,10,11]. As a consequence of this, a number of global, regional, and national initiatives have recently been implemented to promote responsible use of antimicrobials and to curb excessive AMU in animal production [12,13,14,15,16].

In the European Union (EU), a supranational system to monitor AMU in both humans and animals across EU member states has become a reality [17]. A 2014 joint European Centre for Disease Control/European Food Safety Agency/European Medicines Agency surveillance report estimated that, across 28 EU member states, 8927 tonnes of antimicrobial active ingredients were used for animals, compared with 3821 tonnes used for medical purposes [18]. In the USA, antimicrobials used in food animal production accounted for 70% of total antimicrobial consumption in 2014 [10].

The World Health Organization has projected a global increase in meat production from 218 million tonnes in 1999 to 376 million tonnes in 2030, with relatively greater increases in developing countries [19]. The amounts of antimicrobials aimed at animal production worldwide have been forecast to increase by 67% from 2010 to 2030, mostly driven by increased demand for animal protein and intensification of farming systems in low- and middle-income countries (LMICs) [20], although there is considerable uncertainty around the magnitude of this increase. Very little is known about what food animal species are the target of highest levels of AMU in LMICs, while data from high-income countries (HICs) are far from comprehensive. Because of this, international technical agencies have set up initiatives aimed at monitoring AMU/AMR in animal production with a focus on LMICs [21,22].

Measuring AMU in animal production may address different objectives: monitoring AMU over time, setting benchmarks to promote AMU reductions, and investigating associations between AMU and AMR. However, because AMU can be measured using a large diversity of metrics, posing a considerable difficulty to the comparability of data across studies [17]. In addition, limitations in resources and research capacity typical of many LMIC countries represent an additional challenge [23].

In this article, we reviewed and summarized peer-reviewed original research on AMU in terrestrial food animal production worldwide. The aims were as follows: (1) to document methodologies and metrics used to quantify AMU; and (2) to compile qualitative (i.e., prevalence of usage of specific antimicrobials and antimicrobial classes) and quantitative (amounts of antimicrobial active principle), identifying those food animal species (pigs, poultry, or cattle) at highest risk of AMU. We extracted all raw data and metrics reported in these studies, discussed the limitations of the methodologies used, and documented data gaps in LMICs. We hope that this review helps to encourage further harmonization of methodologies aiming at measuring AMU and achieving AMU reductions in animal production globally.

## 2. Materials and Methods

### 2.1. Article Selection

The ‘ISI Web of Knowledge’ engine (Clarivate Analytics, Philadelphia, PA, USA) [24] was used to search for original scientific articles published in English over the period January 1998 to April 2018. The following terms were used to search publications with titles using the following keywords: (antimicrobial* OR antibiotic*) AND (use* OR usage* OR consumption* OR amount* OR quantity*) AND (animal* OR livestock* OR swine* OR pig* OR poultry* OR chicken* OR cattle* OR dairy* OR beef*)]. A wildcard “*” was used to find plurals and word variants, and “multiple terms” used to find similar concept according to the website guidelines [24]. All retrieved records were saved for further review. Publications not reporting original research data, or written in languages other than English were further excluded. Publications containing AMU data in the abstract were selected and their full content was reviewed. Publications were broadly classified by the country where the research took place, and further categorized into whether they were carried out in a LMIC or a high-income country (HIC), based on the World Bank country classification for 2016 [25].

### 2.2. Data Extraction

From each selected publication, the following information was compiled as separate records (data points): (1) country of study; (2) year; (3) study unit (farm/veterinarian/veterinary prescriptions/sales data); (4) number of study units; (5) animal production type: level 1 (species), cattle, poultry, swine, all species combined; level 2, beef cattle, dairy cattle, calves, heifers, broilers, layer chickens, turkeys, weaners, finishing pigs, adult pig/sows; (6) observation period (in months); (7) purpose of usage (non-specified/prophylactic/therapeutic/growth promotion); (8) route of administration (oral/water/feed/injectable/intra-mammary); and (9) source of data in the original publication.

The qualitative data included the reported ‘prevalence of use’ of antimicrobials/antimicrobial classes, or the relative distribution of antimicrobials sold. Quantitative data indicated the amounts used reported, in addition to the relevant expression units. All data were entered as single records (‘data points’) in Excel (Microsoft Office). Antimicrobials and antimicrobial classes listed were those included in the World Organisation for Animal Health (OIE) classification: veterinary critically important antimicrobial (VCIA) agents (10 classes); veterinary highly important antimicrobial (VHIA) agents (8 classes); and veterinary important antimicrobial (VIA) agents (8 classes) [26].

### 2.3. Data Analyses

We further analysed AMU data at farm level, and excluded information from studies based on veterinary prescriptions or pharmacies. AMU estimates from the same study, on the same animal species but on different years, different routes of administration, different production phases, or different types of use, were consolidated into a single data point. The usage rate (probability of use per month) (*UR*) was solved from the standard epidemiological formula:

$$P=1-e^{-UR\times t}$$

Therefore,

$$UR=-\frac{\log\;\left(1-P\right)}{t}$$

where *P* is the reported prevalence of usage (cumulative incidence) and *t* is the reported period of observation (months) [27].

The median (and 75% interquartile range) of the reported *UR* for each of the 10 most used classes of antimicrobials were calculated for cattle, poultry, and swine data.

For quantitative studies, the type of numerator, the population at risk, and the mathematical expressions used to quantify AMU were compiled. The data corresponding to different antimicrobials were added up by class (using the metrics reported). Metrics corresponding to animal-time (i.e., the product of the number of animals times the number of observed time units) were converted to ‘doses per 1000 animal-days’ for swine, cattle (dairy, beef), and poultry. The median (and 75% interquartile range) were given. For antimicrobials where the median across studies was 0, the arithmetic mean and the standard deviation was reported. All analyses were carried out using R statistical software (The R Foundation, Vienna, Austria).

## 3. Results

### 3.1. Publications

**Figure 1:**
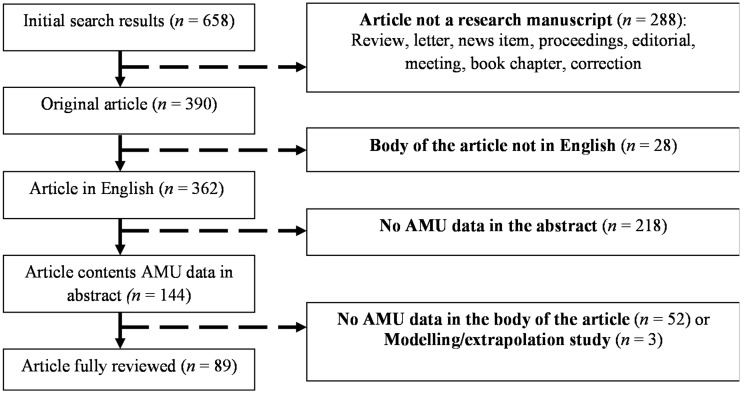
Selection and exclusion criteria for scientific publications on antimicrobial use (AMU) in animal production.

A total of 658 scientific publications were identified using the search terms listed above. Of those, 390 contained original research and 362 were written in English. AMU data (both quantitative and qualitative) was included in the abstract of 144 publications, and all of them were examined. Ninety-two articles contained AMU data within the body of the publication, but three contained extrapolation estimates, rather than survey data [20,28,29], and were thus further excluded, resulting in 89 publications to be reviewed (Figure 1).

The 89 selected studies came from 29 countries (18 of which were classified as HICs and 11 as LMICs, according to the World Bank). Seventy-two (80.9%) studies came from HICs, and 17 (19.1%) from LMICs (8 from Asia, 7 from Africa, and 2 from the Americas). The countries with the highest volume of studies were Canada (11), Denmark (7), Belgium (6), and Germany (5). The studies were classified by publication year, country location, data source, and food animal species (Table 1).

Qualitative (‘prevalence of use’ of antimicrobials/antimicrobial classes, or the relative distribution of antimicrobials sold) and quantitative data (amounts of antimicrobial active ingredient) on AMU were reported in 46 and 60 studies, respectively. Seventeen (19.1%) studies reported both qualitative and quantitative data. Forty-eight percent of studies were published during the recent 2014–2018 period (70.6% of studies from LMICs). Over half (53%) of the studies were performed in Europe, followed by the Americas (23%), Asia (13%), Africa (8%), and Oceania (3%). About 38/47 (80.8%) of European studies reported quantitative AMU data, versus 9/18 (50%) studies from the Americas. A total of 66.3% studies were based on farm survey data, followed by 16.8% based on antimicrobial sales data. The most common animal species investigated were swine and cattle (43.8% studies), followed by poultry (24.7%). Ten percent of studies covered AMU in all species. Of the 17 publications from LMICs, only 7 (41%) reported quantitative data.

### 3.2. Qualitative Data

**Figure 2:**
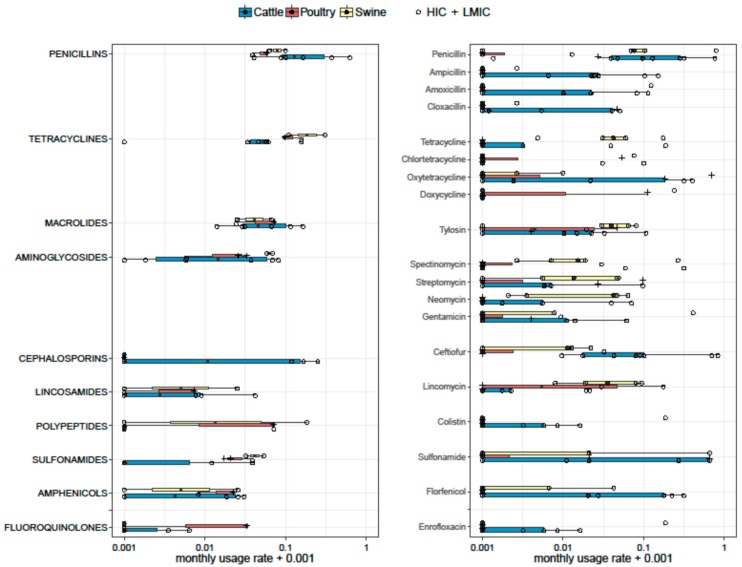
Boxplots representing monthly usage rage (*UR*) of antimicrobials (**Right**) and antimicrobial classes (**Left**). Six, three, and two estimates on antimicrobial classes were available for cattle, poultry, and swine, respectively. Nine, five, and four estimates on specific antimicrobials were available for cattle, swine, and poultry, respectively. The thickness of the boxes reflects the number of studies. HICs—high-income countries; LMICs—low- to middle-income countries.

Forty-six publications reported qualitative AMU data (Supplementary Material S1). These publications generated 50 data points on AMU by class, and 176 data points on use of specific antimicrobials. Data from 19 publications were not further analysed, because either the time frame was not provided, or the data presented reflected the distribution of different antimicrobials used or prescribed, not a prevalence of use. From the remaining 27 publications, 29 data points were compiled, corresponding to use of specific antimicrobials (11) or antimicrobial classes (18). Five data points corresponded to publications from LMICs (from poultry in Vietnam [96,97], Nigeria [105], Tanzania [110], and from cattle in Peru [92]. The usage rate (*UR*) (per month) for the most commonly reported antimicrobials and antimicrobial classes by type of animal production (poultry, swine, and cattle) is displayed in Figure 2.

Two, six, and three estimates on antimicrobial classes were available for swine, cattle, and poultry, respectively. In swine, tetracyclines had the highest *UR* (median 0.209; range 0.108–0.309), followed by polypeptides (0.091; range 0.000–0.183), penicillins (0.080; range 0.062–0.098), and aminoglycosides (0.062; range 0.057–0.067). In cattle, penicillins were the most frequently used antimicrobials in cattle with a median *UR* of 0.130 [inter quartile range (IQR) 0.090–0.320], followed by cephalosporins (0.058 [IQR 0–0.154]), and tetracyclines (0.051 [IQR 0.035–0.059]). The most used antimicrobial classes in poultry were tetracyclines (median 0.095; range 0.095–0.156), followed by macrolides (median 0.071 [range 0.023–0.071]), polypeptides (median 0.069 [range 0.0–0.069]), and penicillins (median 0.057 [range 0.037–0.057]).

Five, nine, and four estimates on specific antimicrobials were available for swine, cattle, and poultry, respectively. Among studies reporting individual antimicrobials in pig farms, the highest *UR* corresponded to penicillin (median 0.075 [IQR 0.068–0.790]), tetracycline (0.041 [IQR 0.040–0.059]), neomycin (0.041 [IQR 0.003–0.046]), and tylosin (0.039, [IQR 0.029–0.063]). In cattle, penicillin was the most used antimicrobial (median 0.096 [IQR 0.039–0.291]), followed by ceftiofur (0.079 [IQR 0.013–0.40]), ampicillin (0.021 [IQR 0–0.060]), and sulphonamides (0.020 IQR [0–0.66]). In chicken farms, the most common antimicrobials used were doxycycline (0.056 [IQR 0–0.605]), followed by tiamulin (0.037 [IQR 0–0.90].

### 3.3. Quantitative Data

Accurate quantification of AMU in animal production requires the integration of two magnitudes, a ‘numerator’, and a ‘population at risk’ denominator (or ‘target population’). The ‘numerator’ indicates the quantities of antimicrobial agent administered (farm surveys), prescribed (survey of veterinary practices), or sold (studies based on sales), in terms of the weight of antimicrobial, the number of animals treated, the number of treatment courses, or the number of animal daily doses. The ‘population at risk’ can be expressed as number of animals (expressed as animals produced, or a ‘stationary’ population census), bodyweight of animals (at slaughter or treatment), or ‘animal-time’ (the product of the number of animals times the number of observed time units) (Table 2).

The most common quantitative metric was the ‘animal daily dose’ (ADD) [47,54,81], or a related expression such as the used daily dose (UDD) [54,57], the prescribed daily dose (PDD) [62], the animal daily dose *x* (ADD*_x_*) [60,61], and the used course dose (UCD) [63]. In conjunction with an animal-time denominator, data on doses can be presented as a ‘treatment incidence’, which can be interpreted as the fraction of time over which animals are under treatment [49].

Thirty-two out of 60 studies reported AMU in animal daily doses related to animal-time, followed by studies reporting weight of antimicrobials related to the following: weight of animal at time of treatment (10), weight of animal production (6), animal-time (4), and number of animals produced (4). Five studies included quantitative AMU data, but the authors did not relate these to a population at risk. The formulae and calculations used in each publication are described in Supplementary Material S2. These 60 studies generated 939 data points related to total AMU use (528), AMU by class (310), and use of specific antimicrobials (108) (Supplementary Material S3). Only 7/60 (11.7%) studies were performed in LMICs.

**Figure 3:**
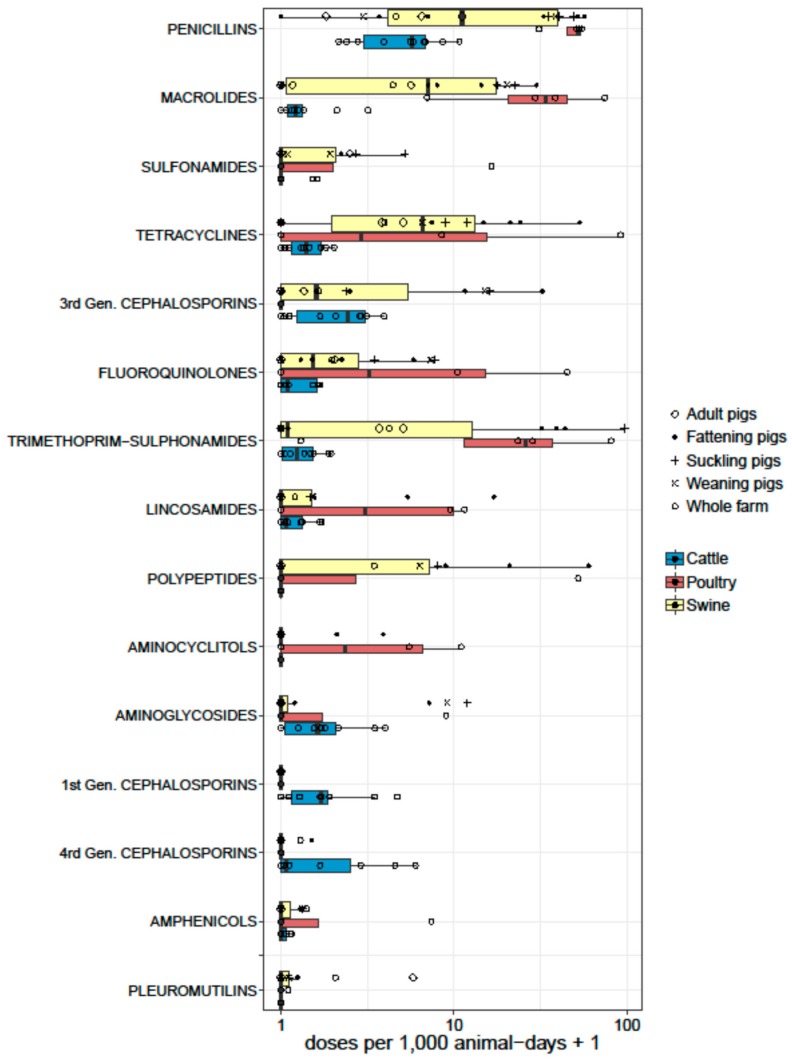
Boxplots representing the summary of AMU by antimicrobial classes from studies reporting quantitative data as doses (per 1000 animal-days) in swine (15), dairy (10), and poultry (4) farms. The thickness of the boxes reflects the number of studies.

Data from studies reporting animal daily doses were standardized as ‘doses per 1000 animal-days’ (equivalent to ‘daily doses per 1000 animals’). Seventeen studies (all from European countries) reported AMU data in swine using these units. Two studies reported partial data (AMU in feeds only) [55,62]. Of the remaining 15 studies, eight reported ‘overall’ AMU on farms [45,47,50,51,54,61,67,116], whereas 7 reported AMU for specific age groups (sows, fattening pigs, suckling pigs, etc.) [32,46,49,56,57,66,68,117] (Figure 3). Across studies, pigs received a median of 40.2 doses per 1000 animals per day (or per 1000 animal-days) [IQR 8.5–120.4]. However, there were differences depending on whether the figures quantified overall (or average) farm AMU, or usage targeted to specific age groups within farms. Data from four studies reported a median of 134.2 [IQR 79.7–134.5] doses per 1000 pig-days for suckling piglets [57,58,70], 8.5 [range 7.9–30.4] to sows/adult pigs [47,57,70,117], and 29.6 [IQR 17.0–34.9] to fattening/finishing pigs [46,50,57,58,68,70]. In decreasing order, the following antimicrobials were given: penicillins (median 10.1 [IQR 2.7–39.7]), trimethoprim-sulphonamides (median 0.10; [IQR 0–31.2]); tetracyclines (median 5.6; [IQR 0–13.8]); macrolides (median 6.1 [IQR 0.16–16.7]); polymyxins (median 0 [IQR 0–7.1]); third generation cephalosporins (median 0.6 [IQR 0–10.6]), aminoglycosides (median 0, [IQR 0–0.2], mean 1.7; SD ± 3.5); and lincosamides (0 [IQR 0–0.5], mean 1.5; SD ± 4.0). Other antimicrobials were used less than 1 mean dose per 1000 pig-days. Antimicrobials in pigs were predominantly administered through the oral route, rather than through the parenteral route [50,54].

Thirteen studies reported dose-based data from dairy farms. All studies came from Europe, except one each from Argentina [93], the USA [91], and Canada [85]. One study reported AMU in heifers before calving [69], and another one reported AMU to treat mastitis [91] exclusively. One study reported separate data for calves, heifers, and dairy cows [63]. The remaining 10 studies reported overall farm AMU (Figure 3). The median number of doses reported in adult cattle was 10.0 doses per 1000 cow-days [IQR 5.5–13.6]. The most used antimicrobials were as follows (in decreasing order): penicillins (median 4.7 [IQR 1.8–5.8]); third generation cephalosporins (median 1.4 [IQR 0.1–2.1]); first generation cephalosporins (median 0.7 [IQR 0.1–0.9]); fourth generation cephalosporins (median 0.1 [IQR 0–1.9]); and aminoglycosides (median 0.6 [IQR 0–1.1]). Five publications reported AMU data as dose-based units in poultry, including three from Europe [51,52,67], one from Canada [84], and one from Vietnam [96]. One of the European studies only reported total use data [51], and data from the remaining four studies are shown in Figure 3. Except the study from Vietnam, which included small- and medium-scale chicken farms, other studies reported data from industrial broiler farms. The median AMU reported was 138 daily doses per 1000 chicken-days [IQR 91.1–438.3]. The Canadian study included in feed antimicrobial growth promoters (AGP) bacitracin and streptogramins, whereas the Vietnamese study did not. AGPs were banned in Europe at the time of the two other studies reported. The most commonly reported antimicrobials were penicillins (median 51.1 [IQR 40.1–52.9]), macrolides (median 33.0 [IQR 17.3–55.4]), trimethoprim-sulfonamides (median 25.0 [IQR 11.4–53.7]), tetracyclines (median 3.8 [IQR 0–49.1]), and fluoroquinolones (median 4.8 [IQR 0–26.9]). Only three studies reported dose-based metrics in beef cattle, of which two reported AMU in veal production in the Netherlands and Belgium [51,67]. A study on beef farms from Canada reported a range of 3.3 to 10.7 per 1000 animal days depending on the type of farm; highest in cow-calf farms, and lowest in mixed feedlot and cow-calf farms. The antimicrobials most commonly given were tylosin (oral) and tetracyclines (injectable) [81].

A number of studies reported AMU related to weight of animal at treatment, standardized as ‘population correction unit’ (PCU) [50,60,61,64,70,74,84,113,114]. One PCU is equivalent to 1 kg of animal body mass at the time of treatment, which is set for each species (i.e., 1 kg for broilers, 65 kg for pigs, and 425 kg for cattle). A similar standardized measure is the LU (‘livestock unit’). One LU was considered to be equivalent to 500 kg of animal biomass (i.e., one adult cow corresponds to ~1 LU, one fattening pig to ~0.15 LU, and one layer hen to ~0.004 LU) [59]. In a recent study, Danish researchers have proposed the use of an ‘adjusted population correction unit’ (APCU), which combines the PCU with the lifespan of the species treated, in order to reflect selection pressure of the antimicrobial over a kilogram of animal per unit time. Calculations using APCU demonstrated that PCU overestimated usage in short-living animal categories (i.e., poultry and, to a lesser extent, pigs), but underestimated AMU in long-living animals (i.e., cattle) [117].

## 4. Discussion

Here, we reviewed 89 studies on AMU in animal production published in English since 1998. In spite that LMICs are home to 84.2% of the global world population, only 17 (19%) publications came from such countries. This imbalance should be addressed, especially given that LMICs will account for the highest increase of AMU over coming years [20]. Interestingly, only one publication (from South Africa) was identified among all five BRICS ‘emerging’ economies (Brazil, Russia, India, China, and South Africa) [111]. It is highly likely, although could not be verified, that this somehow reflects a language bias, and some research has probably been published in languages other than English, or that falls outside the reach of the search engine. Most of the publications from LMICs were obtained from ad hoc farm surveys, as national AMU monitoring systems have not yet been established in most countries. A relatively small fraction of studies (7/17) included quantitative data.

Surveys based on a single farm visit may incur in recall biases, because often farmers do not keep records, especially in small-holder farms typical of many LMICs [118]. Although costly, longitudinal study designs where farmers are requested to keep records and/or antimicrobial product containers can potentially yield more accurate data than unannounced ‘one-off’ visits. However, there is also a risk that farmers may change their behavior or not provide accurate data, the latter being possible if farm visits are carried out by veterinary authorities that are perceived to negatively judge farmers’ AMU practices.

Longitudinal study designs may allow insights into the seasonality of disease [57] and repeated behavior of consumption over time (especially when consecutive cycles of production are investigated) [65]. Such studies may also shed insights into treatment practices for different diseases or types of animal [46,83]. Finally, they may also allow to identify production types, farm sizes, and animal groups at higher risk of usage [45,116], as well as problems with over- and under-dosing [50,54,60,74]. Because longitudinal on-farm surveys are time-consuming and require considerable farmer commitment, they may be affected by a low response rate, limiting their representativeness [50]. In situations where there is a vast diversity of antimicrobial products, but the prevalence of use of each individual product is low, a small sample size may result in a 0 median [51,82], making results difficult to interpret. It would thus be preferable to report the mean and its associated standard deviation. The EU has recently issued recommendations on farm sampling strategies to investigate AMU at species level. These largely depend on the complexity and the size of the country. In the most complex situations (i.e. large countries with high heterogeneity of farming systems), a two-step cluster sampling procedure is recommended. It involves first, randomly selecting regions within the country (clusters), followed by stratification by farm type within each region, and systematic random sampling of farms with a selection probability proportional to their size. The EU also provides recommendations on required sample sizes [119]. In addition, the AACTING initiative aims to provide specific guidelines on monitor AMU at farm-level to monitor antimicrobial stewardship [120].

A number of publications (*n =* 10, of which 5 were from LMICs) reported prevalence of usage without providing a time frame, making interpretation difficult, because usage is dependent on the observation period. A further difficulty in interpreting prevalence of usage data is that in the studies reviewed, no information was provided as to whether antimicrobials were administered to whole flock/herds, or to individual animals. This is particularly relevant in large animal farming (i.e., pigs, ruminants), where individual treatment is common.

None of the studies from LMICs, except one from South Africa [111], included estimates on national sales. Sales data alone does not allow insights into species and production types at highest risk of use. However, if comprehensive, they can be useful to monitor general trends over time, provided that animal production figures remain stable. AMU data collated by national surveillance systems can be used to measure the impact of large-scale interventions, as performed in Norway and Switzerland after the EU compulsory withdrawal of AGPs [33,62], or changes in AMU over time due to the incursion of epidemics [44,49]. In recent years, the EU has implemented joint monitoring of AMU in humans and animals, although the data are mostly reported for all food animals combined [18]. Quantitative data on AMU in specific production types coupled with AMR data may potentially allow the elucidation of the relationship between AMU and AMR [119]. For countries with a considerable fraction of animal production aimed at the export market, it is imperative to include export data in the calculations [47].

As antimicrobials’ active ingredients vary considerably in their potency, the use of dose-based metrics results in more fair comparison between antimicrobials. However, there is no universally accepted dose standards, as these vary by country, species, route of application, and indication [117]. Even if doses are standardized, estimating the number of doses from gross amounts of active ingredient is challenging because animals (especially poultry and pigs) may increase their body size over the production cycle for a factor of 50–100. For oral formulations (often given for flock/herd treatment), the feed and water intake needs to be estimated [80,96], and these data are rarely collected in small-holder farming systems typical of many LMICs. In situations when records are available, it is possible to contrast actual with theoretical use (UDD_animal_/ADD_animal_ or UDD_kg_/ADD_kg_ ratios), and thus estimate the magnitude of over/under-dosing [54,61]. The change of technical specifications of doses may also lead to overall changes in AMU estimates, as shown in Denmark [47]. Comparing dose-based data (i.e., animal daily doses) across studies may present difficulties, because some report overall farm summaries, whereas others report AMU for specific subgroups (i.e., sows, piglets, calves).

In studies where weight and dose-based measures have been compared, some discrepancies have been found for some antimicrobials. For example, doses of tetracycline typically involve higher weights than polypeptides [57,81], fluoroquinolones, and cephalosporins [58]. Recently, the EU has standardized animal daily doses to encourage harmonized reporting across EU member states (termed defined daily dose for animals (DDDvet) [121].

For calculations at national level, animal-time denominator metrics should also take into account the length of empty periods on farms [67]. The definition of denominators based on weights at slaughter is challenging, especially because for long-living animals (i.e., dairy cows, sows, boars), only a small fraction of the standing population of these animals is slaughtered annually. This has been circumvented by using biomass data based on slaughter weight of animals for short living species (poultry, fattening pigs) and standing populations for long-living animals [104]. AMU has also been related to animal produce beyond meat (i.e., eggs and milk) [101]. Estimates of AMU related to food product could be used to define antimicrobial footprints to encourage responsible AMU in food animal production [103].

The European Union countries have agreed on the values assigned to PCU for animal species, which are used to standardize denominator data. However, animal production across the world is highly diverse, and this would require the definition of specific PCU values depending on the production systems. For example, the final slaughter weight of a traditional chicken in southern Vietnam is 1.5–2.2 kg, whereas a typical broiler chicken may reach 2.6 kg. These values, as well as the variability in prescribing practices, are likely to affect the weight of animals at time of treatment.

Our review suggests a great variability in levels of AMU, between countries and species, as well as across age/production groups within species. Overall, AMU expressed as doses per unit of animal-time was highest in broiler production, followed by pig and dairy. An exception to this was a study from Belgium, where treatment incidence was higher in pig than in broiler production [51]. A study from Japan using estimates related to weight of animal production suggests that the amounts of antimicrobials used to produce 1 kg of pork far outweigh the amounts used to produce 1 kg of broilers or cattle [104]. This is likely to reflect the longer production cycle of pigs versus broilers (6 months vs. 1–1.5 months). Although adult cattle used generally fewer doses of antimicrobials per unit time, the use of critically important antimicrobials such as broad spectrum *β*-lactams and cephalosporins to treat mastitis infections is of great concern [30,31,83]. A considerable target of AMU in dairy cattle is the treatment of clinical mastitis and dry cow therapy [93]. We would like to highlight the lack of studies on AMU in poultry breeding flocks, laying flocks, and hatcheries worldwide. In some countries, it is common practice to dip or inject hatching eggs with antimicrobials to reduce the incidence of early infections [122].

This review confirmed a considerable deficit of studies on AMU from LMICs. Because of these data limitations, it cannot be concluded whether farms in LMICs are at higher or lower risk of AMU than their HIC counterparts. Also, it not clear to what extent animals in small-scale farms are raised using more or less antimicrobials than animals raised in larger (i.e., industrial) farms. There is conflicting evidence on this. One study from Vietnam showed higher levels of AMU in small- compared with medium-scale chicken farms [97]. Another study from the same country showed that pork, beef, and chicken meat samples purchased from wet markets were more commonly contaminated with antimicrobial residues than samples purchased from supermarkets. As supermarkets generally source their meat from industrial farms, this suggests higher levels of AMU in smaller farms [123]. However, another study on Thai pig farms reported the higher levels of antimicrobial usage in medium farms compared with small farms [99]. Although income limitations among farmers in LMICs may theoretically result in lower levels of AMU, in practice this may be offset by a higher incidence of infectious diseases, easier access to veterinary drugs, limited veterinary services, and generally looser legislative enforcement [124,125]. It is hoped that as more research/surveillance data on AMU in LMICs becomes available, this will become clearer.

## 5. Conclusions

We reviewed English-language scientific literature covering metrics and data pertaining to AMU in terrestrial animal production. Examination of these data indicates a considerable diversity of methodologies, as well as biases towards data from HICs and a concomitant data deficit from LMICs. Given the challenges posed by the variability of animal production systems, it would seem a priority to encourage the performance of on-farm surveys, and to recommend as a priority the collection of data as gross amounts (weight) of antimicrobial active ingredient by production system, and to further integrate these with production data collected at country level. The quantification of AMU using dose-based metrics should be carried out after the baseline data become available, but this requires standardization of dose definitions. In terms of treatment incidence, usage in poultry production is the highest, followed by AMU in swine and cattle production. We hope these data encourage the further investigation of AMU especially in LMICs with the aim of reducing the pressing threat of AMR worldwide.

## Figures and Tables

**Table 1:** Summary of 89 publications on antimicrobial use (AMU) stratified by year of study, country location, study design, and animal species, stratified by type of data (quantitative and/or qualitative) and type of country according to the World Bank income classification (2016). Individual studies are identified in the footnote (countries classified as low- to middle-income countries (LMICs) by the World Bank in 2016 are underlined). HICs—high-income countries.

Category	Sub-Category	Number of Studies (%)								
		HICs			LMICs			All Studies		
		Qualitative (*n =* 32)	Quantitative (*n =* 53)	All Types (*n =* 72)	Qualitative (*n =* 14)	Quantitative (*n =* 7)	All Types (*n =* 17)	Qualitative (*n =* 46)	Quantitative (*n =* 60)	All Types (*n =* 89)
**Year of publication**	2014–2018	9 (28)	26 (55)	31 (43)	10 (72)	6 (86)	12 (70)	19 (41)	35 (59)	43 (48)
	2009–2013	8 (25)	13 (24)	19 (26)	2 (14)	1 (14)	3 (18)	10 (22)	14 (23)	22 (25)
	2004–2008	12 (38)	8 (15)	17 (24)	2 (14)	0 (0)	2 (12)	14 (30)	8 (13)	19 (21)
	1998–2003	3 (9)	3 (6)	5 (7)	0 (0)	0 (0)	0 (0)	3 (7)	3 (5)	5 (6)
Country location *	Europe	13 (41)	38 (73)	47 (65)	0 (0)	0 (0)	0 (0)	13 (28)	39 (65)	47 (53)
	Americas	17 (53)	9 (17)	18 (25)	2 (14)	1(14)	2 (12)	19 (42)	10 (16)	20 (23)
	Asia	1 (3)	2 (4)	3 (4)	6 (42)	5 (72)	8 (47)	7 (15)	7 (12)	11 (12)
	Africa	0 (0)	0 (0)	0 (0)	6 (42)	1(14)	7 (41)	6 (13)	1 (2)	7 (8)
	Oceania	1 (3)	2 (4)	4 (6)	0 (0)	0 (0)	0 (0)	1 (2)	3 (5)	4 (4)
**Study design**	Farm survey	27 (84)	33 (62)	48 (67)	11 (79)	6 (86)	13 (76)	38 (83)	38 (60)	59 (66)
	Sales data	1 (3)	15 (28)	15 (28)	0 (0)	1 (14)	0 (0)	1 (2)	15 (24)	15 (16)
	Veterinarian survey	4 (13)	6 (11)	10 (19)	1 (7)	0 (0)	1 (6)	5 (11)	7 (11)	11 (12)
	Pharmacy survey	0 (0)	2 (4)	2 (4)	2 (14)	0 (0)	3 (18)	2 (4)	3 (5)	5 (6)
**Animal species**	Swine	11 (31)	25 (47)	36 (50)	3 (19)	1 (11)	4 (23)	14 (30)	26 (43)	39 (44)
	Cattle	20 (56)	23 (43)	36 (50)	3 (19)	2 (29)	3 (18)	23 (50)	27 (45)	39 (44)
	Poultry	5 (14)	11 (21)	13 (18)	7 (44)	5 (71)	9 (53)	12 (26)	16 (27)	22 (25)
	Combined data	0 (0)	5 (9)	5 (7)	3 (19)	1 (11)	4 (23)	3 (7)	6 (10)	9 (10)

* Europe, qualitative (13): Austria [30], Belgium [31], Germany [32], Norway [33], Italy [34,35,36], Spain [37,38], Finland [39], France [40]; UK [41,42], several EU countries [42]; Europe, quantitative (39): Denmark [43,44,45,46,47,48,49], Belgium [31,50,51,52,53,54], Germany [32,33,55,56,57,58,59] Austria [59,60,61,62], Switzerland [63,64], Netherlands [65,66,67], Sweden [68,69], France [40,70], Norway [33], Ireland [71,72], Italy [36], several EU countries [5,73], UK [74]; The Americas, qualitative (19): Canada [75,76,77,78,79,80,81,82,83,84,85], USA [86,87,88,89,90,91], Peru [92], Argentina [93]; The Americas, quantitative (9): Canada [75,80,81,82,83,84,85], USA [91,94], Argentina [93]. Asia, qualitative (7): Vietnam [95,96,97], Cambodia [98], Thailand [99], Japan [100], Iran [101]; Asia, quantitative (7): Vietnam [96,97,102], Thailand [103], Japan [6,104], Iran [101]. Africa, qualitative (6): Nigeria [105,106,107,108], Cameroon [109], Tanzania [110]; Africa, quantitative (1): South Africa [111]. Oceania, qualitative (1): Australia [112]; Oceania, quantitative (3): New Zealand [113,114,115].

**Table 2:** Classification of 60 publications reporting antimicrobial use (AMU) quantitative data by the type of metrics used and animal production types. Studies performed in LMICs are underlined. The number of publications reporting using those metrics is given in parentheses.

		Type of Animal Production (N)						All Studies (*N*)
	Population at Risk	Dairy	Beef	Cattle (Unsp.)	Poultry	Swine	Total Use	
Weight of antimicrobial	Animal-time	[63] (1)	[81] (1)	-	[97] (1)	[94] (1)	-	(4)
	No. animals produced	[69] (1)	-	-	[80,97] (2)	[47] (1)	-	(4)
	Weight of animal production	[104] (1)	[104] (1)	[101] (1)	[101,102,103,104] (4)	[44,102,104] (3)	-	(6)
	Weight of animal at treatment	[74,113] (2)	-	[64] (1)	[84] (1)	[60,61,64] (3)	[5,70,114] (3)	(10)
	Weight of animal time	[63] (1)	-	-	-	-	-	(1)
	No population at risk	[71,115] (2)	-	[5,43] (2)	[5] (1)	[5,43] (2)	[111] (1)	(5)
No. animals treated	Animal-time	-	-	-	-	[75] (1)	-	(1)
	No. animals produced	-	-	-	[33] (1)	-	-	(1)
No. treatment courses	Animal-time	[63] (1)	-	-	-	-	-	(1)
	No. animals produced	-	[40,41] (1)	-	-	-	-	(1)
No. daily doses	Animal-time	[30,31,53,63,65,69,72,74,82,83,85,91,93] (13)	[51,67,81] (3)	[56] (1)	[51,52,67,84,96] (5)	[32,45,46,47,49,50,51,54,55,56,57,60,62,66,67,68,116] (17)	-	(32)
	No population at risk	[58] (1)	-	-	-	[58] (1)	[59] (1)	(2)
No. studies		(18)	(5)	(5)	(13)	(27)	(5)	(60)

## Supplementary Materials

The following are available online at http://www.mdpi.com/2079-6382/7/3/75/s1.
