# Supplementary material for: Antimicrobial Usage in Animal Production: A Review of the Literature with a Focus on Low- and Middle-Income Countries
Source: Antibiotics (Basel). 2018 Aug 15;7(3):75. doi: 10.3390/antibiotics7030075 (PMC6164101; doi:10.3390/antibiotics7030075)
Supplement: Supplementary file 1 [file antibiotics-07-00075-s001.zip › antibiotics-326610-Supplementary Material S2 For Publication.docx]

Review

Antimicrobial Usage in Animal Production:
A Review of the Literature with a Focus on Low-
and Middle-Income Countries

Nguyen V. Cuong ^1^, Pawin Padungtod ^2^, Guy Thwaites ^1,3^ and Juan J. Carrique-Mas ^1,3,^*

^1^ Oxford University Clinical Research Unit, 764 Vo Van Kiet, District 5, Ho Chi Minh City, Vietnam; cuongnv@oucru.org (N.V.C.); gthwaites@oucru.org (G.T.)

^2^ Emergency Center for Transboundary Animal Diseases, Food and Agriculture Organization of the United Nations, Green One UN House Building, 304 Kim Ma, Hanoi, Vietnam; Pawin.Padungtod@fao.org

^3^ Centre for Tropical Medicine and Global Health, Nuffield Department of Medicine, Oxford University, Old Road Campus, Headington, Oxford OX3 7BN, UK

***** Correspondence: jcarrique-mas@oucru.org

Received: 24 June 2018; Accepted: 10 August 2018; Published: date

**Table S2.** Calculations used for different types of unit used in quantitative studies.

| Ref. | Expression | Calculations | Definitions |
| --- | --- | --- | --- |
| [30] | nDDDvet | $nDDDvet=\frac{amount active substance (mg)}{DDDvet for that antimicrobial substance}$  $nDDDvet/cow/year=\frac{\mathrm{nDDDvet}}{Production days x Standardised liveweight (500kg)} x 365$  $nDDDvet per cow per year \left( \mathrm{intramam} \right): =\frac{\mathrm{nDDDvet}}{Production days x Standardised liveweight (500kg)} x 365$ | DDDvet = Defined daily dose for animals |
| [31] | ATI = nDDDAs/1,000 cow/day | $ATI=\frac{Total amount of active subtance or combination of active substances (mg)}{\mathrm{DDDA}\left( \frac{\mathrm{mg}}{cow-day} \right)x cow-days}$ | ATI = Antimicrobial treatment incidence  DDDA = Defined Daily Dose Animal= average daily on-label dosage (mg/kg) multiplied by the approximate weight of an adult dairy cow (600kg) |
| [32] | TF=nUDD/farm size | nUDD = No. animals treated x No. days treated | TF = Treatment frequency  UDD = No. used daily doses |
| [33] | % broilers treated | $\% broilers treated =\frac{\left( \frac{mg antibacterial drugs sold per year}{DDD poultry (per kg live weight)} \right)}{Weight of broilers x Days of treatment x No. broilers produced per year}x 100$  Weight=0.8kg; Days of treatment=3 | DDD = Defined daily dose |
| [40] | NTPC_pdd_ew | NTPC_pdd_ew=No. of treatments per calf based on prescribed daily dosagesand an estimated weight |  |
| [45] | TI rate | $TI rate =\frac{No. of ADDs}{100 slaughter pig-days}$ | ADD = Animal daily dose |
| [46] | nADD15/weaner produced; nADD50/fattening pig produced;  nADD200/sow-year |  | The standard doses, ADD200 for sows, ADD50 for slaughter pig, and ADD15 for weaner pigs, were defined as the doses for 200, 50, and 15 kg live weight, respectively, assumed as the average weights at treatment in each age-group |
| [47] | nADD different calulations) | $nADD=\frac{Amount of antimicrobial sold}{Dosage per kg body weight \left( b \right) x standard weigth (c)}$  b=ADD value according to VetStat, DANMAP or DVFA; c=Standardised VetStat values for weight at treatment: growers (15kg); finishers (50kg); pre-weaning pigs, sows, boars and gilts (200kg) |  |
| [48] | ND25 (or nDADD25)/pig produced | $ND25 =\frac{[ND\left( \mathrm{sows}\&\mathrm{piglets} \right)+ ND(weaners) + (1+Q/(N- Q))*ND(finishers)]}{25 x N}$  ND=Annual No. of DADDs used in sows/piglets, weaners and finishers, Q=Number of pigs exported at around 30kg bw; N=Total number of pigs produced, incl. export; and ’25’, the chosen weight (kg) of a standard pig | DADD25 = The standard dose for treatment of 25 kg body weight |
| [49] | nADDkg/pig/day | nADDkg *herd, day* = ADDkg*herd; prescribed*/ndays *herd; prescription period*  nAADkg *herd;month* = $\sum_{date=1}^{n} \mathrm{AADkg}herd;day$  n pig-days *herd;month* = $\sum_{date=1}^{n} \mathrm{pigs}herd;day$  nADDkg/pig/day = ADDkg *herd;month*/pig-days *herd;month* | ADDkg *herd; prescribed* = Amount of antimicrobials measured as ADDkg prescribed to the herd at the initial day of the prescription period; ndays *herd; prescription period* = No. days between the day of the actual prescription and the day of the subsequent prescription;  npig-days *herd; month* = No. of pig-days at risk for antimicrobial treatment every month.  nADDkg/pig/day = Amount of antimicrobials used per pig-day (ADDkg/pigday) within a herd each month |
| [50] | TI-ADDpig/1,000 animal-days | $TI=\frac{Amount of antimicrobial sold}{\mathrm{ADD}\left( \frac{\mathrm{mg}}{\mathrm{kg}} \right)x No. days at risk x kg pig}$ | TI-ADD pig = Treatment incidence of Animal Daily Doses (number of pigs per 1,000 that is treated daily with one ADDpig |
|  | TI-UDDpig/1000 animal-days | $TI=\frac{Amount of antimicrobial sold}{\mathrm{UDD}\left( \frac{\mathrm{mg}}{\mathrm{kg}} \right)x No. days at risk x kg pig}$ | TI-UDDpig = Treatment incidence of Animal Used Daily Doses (Treatment incidence of Animal Daily Doses (number of pigs per 1000 that is treated daily with one UDDpig |
| [51] | TI-DDDA per 1,000 animal/day | $TI-DDDA=\frac{active ingredient \left( \mathrm{mg} \right)}{\mathrm{DDDA}\left( \frac{\mathrm{mg}}{\mathrm{kg}} \right)x Number of days at risk x kg of animal} x 1,000$ | TI-DDDA = No. of animals per 1,000 that are treated daily with one defined daily dose animal (DDDA) (mg/kg) |
|  | TI-UDDA per 1,000 animal/day | $TI-UDDA=\frac{active ingredient \left( \mathrm{mg} \right)}{\mathrm{UDDA}\left( \frac{\mathrm{mg}}{\mathrm{kg}} \right)x Number of days at risk \left( \mathrm{kg} \right)} x 1,000$ | TI-UDDA = No. of animals per 1,000 that are treated daily with one used daily dose animal (UDDA) (mg/kg) |
| [52] | TI-DDD/ 1,000 animal-day | $TI=\frac{total amount of antimicrobial active ingredient \left( \mathrm{mg} \right)}{\mathrm{DDD}\left( \frac{\mathrm{mg}}{\mathrm{kg}} \right)x Number of days at risk x kg of animal}$x 1,000 | TI-DDD = Treatment incidence of Defined Daily Doses |
|  | TI-UDD/ 1,000 animal-day | $TI=\frac{total amount of antimicrobial active ingredient \left( \mathrm{mg} \right)}{\mathrm{DDD}\left( \frac{\mathrm{mg}}{\mathrm{kg}} \right)x Number of days at risk x kg of animal}$x 1,000 | TI-UDD = Treatment incidence of Used Daily Doses |
| [54] | TIADDpig = nADDpig/1,000  pigs/day  TIUDDpig = nUDDpig/1,000  pigs/day | $TI-ADDpig =\frac{Amount of antimicrobial drug used (mg)}{\mathrm{ADD}\left( \frac{\mathrm{mg}}{\mathrm{kg}} \right)x No. days at risk x kg pigs at risk}$  $TI-UDDpig =\frac{Amount of antimicrobial drug used (mg)}{\mathrm{ADD}\left( \frac{\mathrm{mg}}{\mathrm{kg}} \right)x No. days at risk x kg pigs at risk}$ | ADDpig = Animal daily dose  UDDpig = Used daily dose  TIADDpig = Treatment Incidence based on the ADDpig  TIADDpig = Treatment Incidence based on the UDDpig |
| [55] | Treatment days/animal-quarter | Ungemach *et al.* (2006) |  |
| [56] | nDDay | $n daily doses =\frac{free substance (mg)}{recommended dosage \left( \frac{\mathrm{mg}}{\mathrm{kg}} \right)x Average animal weight}$  $nDDay=\frac{n daily doses}{Population size (Animal-years)}$ | nDDay = Number of daily doses per animal-year.  The average animal weight per species and animal age group was Piglet, 12.5kg; weaner, 25 kg; fattener, 70.2 kg; sow, 220 kg; calf: 80 kg; heifer, 300kg; cow, 600kg, bull, 600kg, and cattle, 600kg |
| [57] | TF | nUDD = No. treated animals x No. treatment days x No. active ingredients  $TF=\frac{\mathrm{nUDD}}{population size (animals-time)}$ | UDD = Used daily dose  TF = The treatment frequency specifies how many days an animal in a herd is treated with one active ingredient on average |
| [60] [61] | nUDD (kg/kg/year) | $UDDkg =\frac{\left( \mathrm{used} \right)active ingredient (mg)}{No. treated animals x standard weight \left( \mathrm{kg} \right) x treatment duration}$ | UDDkg = Used daily dose per kilogram of body mass |
|  | nADD (kg/kg/year) | $ADDkg =\frac{active ingredient (mg)}{No. treated animals x standard weight \left( \mathrm{kg} \right) x treatment duration}$ | Animal Daily dose per kg biomass (ADDkg) = Maintenance dosage per day and per kg biomass for a drug for its main indication in one species |
| [62] | nPDD/animal/year | PDD = Average weight of the animals (kg)* No. of  animals (n)* Treatment period (days) | PDD = Prescribed daily dose (mg ): Amount of active ingredient per prescription |
| [63] | TI-UCDa=  nUCDa/animal/year | $UCDkg (per couse)=\frac{active ingredient (mg)}{standard weight \left( \mathrm{kg} \right)}$  $nUCDa=\frac{\mathrm{nUCDkg}}{standard weight \left( \mathrm{kg} \right)}$  $TI-UCDa=\frac{\mathrm{nUCDa}}{animal-years risk}$ | UCDkg = Used course dose per kilogram  UCDa = Used course dose per animal  TI-UCDa = Treatment incidence of UCDa |
|  | TI-UDD=  nUDD/100 animals/year | $TI-UDD=\frac{\mathrm{nUUD}}{100 animal-years risk}$ | UUD = Used unit doses |
| [65] | ADDD*_th_* | $\mathrm{ADDD}th=\frac{\sum_{i=1}^{n} \left( B_{i} / {ADD}_{i} \right)}{C_{th}}$  $ADDD dry-cow=\left( \frac{PC}{100} \right)x 4 x \left( \frac{365}{CI} \right) x \left( 1-\frac{PR}{100} \right) x \left( 1-\frac{PTT}{100} x 1/4 \right)$ | ADDD*th* = Animal defined daily doses in a given year in a given herd  Bi = Amount of drug bought by or sold to herd h in year t in ml, tubes, or g;  ADD*i* = Animal (cow) defined daily dosage of drug i measured in ml, tubes, or g;  Average cow weight = 600kg; average calf weight: 60kg;  ADDD*dry-cow* = The number of daily dosages (tubes) per cow per year that were used for dry-cow therapy  PC = percentage of cows remaining in a herd that receive dry-cow therapy (all teats);  CI = calving interval of the herd (days); PR = percentage of cows replaced; and PTT = percentage of cows that have 3 teats instead of 4 |
| [66] | No. doses/pig-year |  |  |
| [67] | ADDD/Year |  | ADDD/Year = Animal defined daily doses per year |
|  | ATD/Year |  | ATD/Year = Animal treatment days per year |
| [68] | TI/1,000 pig/day | $TI=\frac{active ingredient \left( \mathrm{mg} \right)}{\mathrm{DDDA}\left( \frac{\mathrm{mg}}{\mathrm{kg}} \right)x Number of days at risk x kg of animal} x 1,000$ | TI = Treatment incidence based on defined daily dose animal (DDDA) |
| [72] | nDDDvet/1,000 animals  nDCDvet/1,000 animals | $nDDDvet per 1,000 animals=\frac{No. tubes sold for lactation use}{\left( \frac{Assigned DDDvet for a tube}{No. lactating cows x 1,000} \right)}$  $nDCDvet per 1,000 animals=\frac{No. tubes sold for lactation use}{\left( \frac{Assigned DCDvet for a tube}{No. lactating cows x 1,000} \right)}$ | DDDvet = Defined daily dose for animals (=1 intra-mammary tube)  DCDvet = Defined course per animals (=3 intra-mammary tubes) |
| [75] | Pigs treated/1,000 pig-days | $Pigs treated per 1,000 pig-days=\frac{No. of initial treatments with antimicrobials x 1,000}{\left[ \frac{\left( No. of pigs at beginning of study \right)+ \left( No. pigs at end of study \right)}{2} \right]x Monitoring period (d)}$ |  |
| [81] | Kg active ingredient/1,000 animal-days |  |  |
|  | nADD/1,000 animals/day | $nADD=\frac{active ingredient (mg)}{labelled daily dose \left( \frac{\mathrm{mg}}{\mathrm{kg}} \right)x standard weigth (kg)}$ | ADD = Animal daily dosages |
| [82] [83] | ADUR = nADD/1,000 cow-days | ADD (g/d) | ADD = Animal-defined daily doses  ADUR = Antimicrobial drug used rate |
| [84] | nDDDvetCA/1,000 chicken-days | $nDDDvetCA=\frac{Antimicrobials in feed \left( g \right)+water \left( \mathrm{mg} \right)+injection (mg)}{\mathrm{DDDvetCAmg}}$ | DDDvetCA = Defined-daily doses using Canadian standards  DDDvetCAmg = Defined-daily doses using Canadian standards for a specified antimicrobial (in mg) |
| [85] | nADD  ADDR  ADUR | ADDR=ADD/No. days (by farm)  ADUR = ADD/100 cow-years | ADD = Animal daily dose  ADDR = Animal daily dose rate  ADUR = Antimicrobial drug use rate |
| [93] | DUa | $\mathrm{DU}a=\frac{(TDa/nLC) or (TDa/nPWC)}{\mathrm{DDDAa}}$  TDa=Reported total dose; nLC=Number of lactating cows; nPWC=Number of weaned calves; DDDAa=Defined daily dose per animal for that compound. | DUa = Number of DDDA per animal per year of a given compound (a) |
| [96] | TI | $TI=\frac{Total amount of antimicrobial administered (mg)}{\mathrm{DDDs}\left( \frac{\mathrm{mg}}{\mathrm{kg}} \right)x No. days at risk x Weight of chickens on farm (kg)}$ | TI = Treatment incidence or No. of chickens per 1,000 that are treated daily with one DDD.  DDD = Defined Daily Dose |
| [97] | g/1,000 animals  produced | $g per 1,000 chickens produced=\frac{Estimated annual usage (g)}{No. chickens in farm x No. chicken cycles per year}$ |  |
|  | mg/animal/week | $mg per chicken/week=\frac{Reported usage over a period (g)}{No. chickens in farm x Weeks of reporting period}$ |  |
| [101] | mg antimicrobial/Kg animal food | $Consumption factor=\frac{Annual amount of active ingredient (mg)}{weight of food annimal produced (kg)}$ |  |
| [116] | nDDDs/1000 animals-*die* | nDDDs/1000 animals-*die* = [Prescribed DDDs/(No. reared animals x Days of observation (365)] x 1,000 | DDD = Defined daily dose |
